# Internal epitope tagging informed by relative lack of sequence conservation

**DOI:** 10.1038/srep36986

**Published:** 2016-11-28

**Authors:** Leonard Burg, Karen Zhang, Tristan Bonawitz, Viktorija Grajevskaja, Gianfranco Bellipanni, Richard Waring, Darius Balciunas

**Affiliations:** 1 Department of Biology, College of Science and Technology, Temple University, Philadelphia, PA 19122, United States.

## Abstract

Many experimental techniques rely on specific recognition and stringent binding of proteins by antibodies. This can readily be achieved by introducing an epitope tag. We employed an approach that uses a relative lack of evolutionary conservation to inform epitope tag site selection, followed by integration of the tag-coding sequence into the endogenous locus in zebrafish. We demonstrate that an internal epitope tag is accessible for antibody binding, and that tagged proteins retain wild type function.

High-throughput experimental approaches such as chromatin immunoprecipitation and immunoprecipitation mass spectrometry rely on strong and highly specific recognition of the protein of interest using antibodies^1^,^2^. Two methods have been widely used to facilitate these experimental approaches. The first is generation of antibodies against the protein of interest. Unfortunately, production of custom antibodies is time consuming and does not always yield antibodies of sufficient quality. Even when satisfactory antibodies are generated, downstream experimental procedures need to be optimized for each new antibody. The second method is to express the protein of interest with a well-characterized epitope such as V5, FLAG or Myc^3^. This enables researchers to use commercially available, validated reagents and protocols for downstream applications. Historically the disadvantage of this method has been that the tagged protein is expressed via an ectopic expression system. Ectopic expression almost inevitably leads to non-physiological levels of expression, resulting in potential artifacts among identified protein-protein or protein-DNA interactions. Furthermore, it is rarely possible to test if the tagged protein matches the functionality of the wild type counterpart at normal cellular concentration.

The limitations of these traditional strategies can be overcome by inserting an epitope-coding sequence into the endogenous locus as is frequently done in yeast^4^. The tag-containing construct remains in the native regulatory environment of the gene, maintaining proper expression level. Recent advances in the application of targeted nucleases, CRISPR/Cas9 in particular, enable efficient introduction of precise changes at specific loci^5^,^6^,^7^,^8^,^9^,^10^,^11^,^12^.

It is technically equally feasible to insert an epitope tag into the N-terminus, the C-terminus, or any other location of the open reading frame. Internal integration of the epitope tag offers an important advantage: frameshift mutations can be isolated in parallel with epitope tagging. Together, these can be used to characterize loss-of function phenotype(s) and to determine if the epitope-tagged protein is functionally wild type.

One major hurdle, however, concerns how to select the precise location for tag integration into a given protein. Intuitively, the epitope tag should be on the “outside” of the protein to be accessible for antibody binding in its non-denatured state. Furthermore, the epitope tag should not significantly disrupt folding of the protein of interest or negatively affect critical protein-protein interactions. We hypothesized that evolutionary conservation can be used to guide internal epitope-tagging approaches. We posit that regions of relatively low conservation are unlikely to be involved in the critical function of the protein. Evolutionary biology further suggests that indels in the protein sequence are most likely to occur in regions which form unstructured “loops” on the surface of a protein^13^. We therefore hypothesized that protein segments of variable length, in addition to relatively low conservation, would be the most suitable targets for internal epitope tagging.

## Results

### Selection of target sites for epitope integration

For our proof-of-principle experiments we selected two zebrafish genes for which mutant alleles had not yet been characterized: *tcf21* and *tbx18*. Both genes are expressed in the developing proepicardial organ. Interestingly, overexpression of *tcf21* results in increased *tbx18* expression, though it is not clear if this is a direct effect^14^. In addition to the proepicardial organ, *tcf21* is expressed in myoblast precursors in the branchial arches^15^,^16^,^17^,^18^, while expression of *tbx18* is most pronounced in the developing pectoral fin bud^19^. In adult zebrafish, *tcf21* is constitutively expressed in the epicardium, and ablation of *tcf21*-expressing epicardial cells leads to loss of the regenerative capacity of the zebrafish heart^20^. In contrast, *tbx18* is not expressed in the adult heart, but it is upregulated in the epicardium in response to injury^21^.

**Figure 1:**
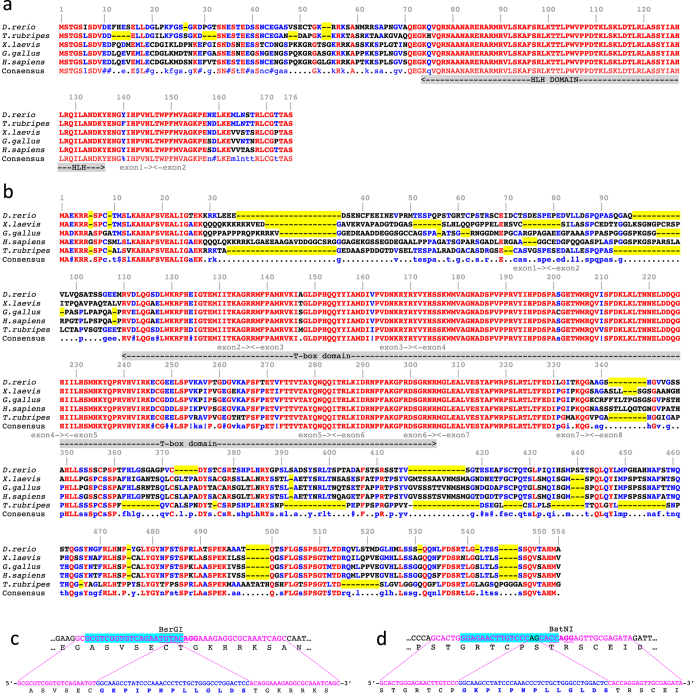
Target site selection for epitope tagging and mutagenesis. (**a)** Alignment of the zebrafish, fugu, Xenopus, chicken and human Tcf21 protein sequences. Amino acids identical between all species are in red, identical between at least three out of five species in blue. Absent amino acids (length variation) are highlighted in yellow. Selected target for tag integration is shown by a blue triangle. (**b)** Alignment of the zebrafish, fugu, Xenopus, chicken and human Tbx18 protein sequences. (**c,d)** DNA sequences of *tcf21* (**c**) *tbx18* (**d**) target sites (top) and repair oligonucleotides (bottom). sgRNA target sequence is highlighted in cyan, PAM sequence is shown in bold, location of the Cas9-induced DSB is shown by a red line, restriction enzyme site is underlined, homology arms are in magenta, and sequence of the V5 tag is in blue.

To identify relatively non-conserved regions we aligned zebrafish Tcf21 and Tbx18 protein sequences with their homologs from fugu, African clawed frog, chicken and human, covering a wide spectrum of vertebrate lineages which diverged over 400 million years ago^22^. We found that Tcf21 protein sequences are extremely highly evolutionarily conserved, with DNA binding domains being nearly 100% identical (Fig. 1a). We also noted a high degree of conservation in the very N- and C-termini: 8/9 N-terminal and 7/8 C-terminal amino acids are identical among the aligned proteins. The overall level of conservation among the Tbx18 proteins is not quite as high as that of Tcf21 (Fig. 1b). However, both N- and C-termini are also highly conserved: the first six amino acids are identical between zebrafish and human, and 6/8 C-terminal amino acids are identical among all five species analyzed. In contrast, the internal part of the protein, except for the T-box DNA binding domain, is much more loosely conserved. The high degree of sequence conservation prompted us to not pursue classical N- or C-terminal epitope tagging. Furthermore, functional domains (apart from the DNA binding domains) of our proteins of interest are not well defined, making it impossible to pursue a domain-based tagging approach such as NLS-tagging^23^. We therefore decided to focus on relative lack of evolutionary conservation as the main guiding principle to select tag integration sites. We also applied two additional criteria for our target sites. First, since we wanted to be able to generate likely null mutants in parallel with epitope-tagged alleles, we selected target sites preceding the DNA-binding domain. Second, to facilitate efficient and straightforward analysis of CRISPR/Cas9 activity as well as genotyping of recovered mutants, we selected target sites either within or near a restriction enzyme site. Based on these criteria, we chose to insert the epitope after Cys51 in Tcf21 and after Ser64 in Tbx18 (Fig. 1c,d).

We selected the V5 epitope for our studies because a single V5 epitope is sufficient for strong antibody binding, a variety of reagents -including anti-V5 coated agarose beads- are commercially available, and the V5 epitope tag has been successfully used for ChIP-Seq experiments in the mouse^24^,^25^. To insert the V5 epitope by homology-directed repair, we decided to provide a single-stranded oligonucleotide template with the epitope-coding sequence flanked by approximately 20 nucleotide-long homology arms similar to the strategy used to integrate a loxP site into a TALEN-induced double strand break^26^.

### Assessment of sgRNA activity and tag integration in injected embryos

**Figure 2:**
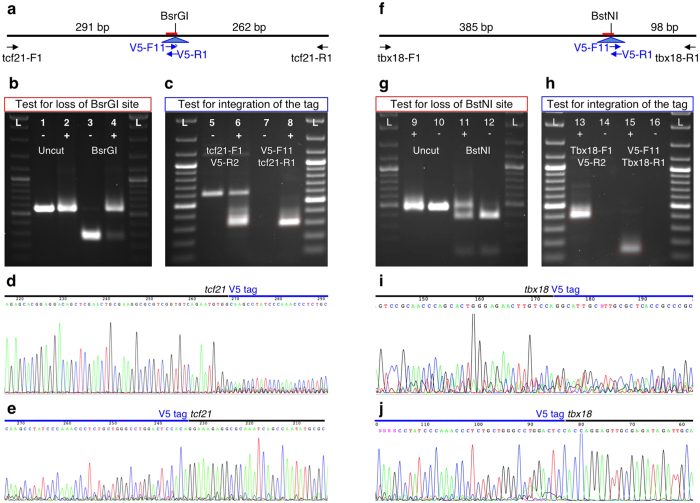
Testing of sgRNA activity and tag integration in injected embryos. (**a**,**f)** Graphic representation of the *tcf21* (a) and tbx18 (f) target loci and different PCR primers relevant for (**b**–**e**). (**b,g)** Testing CRISPR/Cas9 activity by loss of the BsrGI (**b**) and BstNI (**g**) restriction enzyme sites. PCR fragments generated using flanking primers and DNA prepared from embryos injected with appropriate sgRNA and V5-coding oligonucleotide (+) and embryos injected with control sgRNA and V5-coding oligonucleotide (−) were separated by agarose gel electrophoresis either undigested (lanes 1, 2) or after digestion with the appropriate restriction enzyme (lanes 3, 4). On both sides, ThermoFisher Scientific GeneRuler DNA ladder. (**c,h)** Testing for epitope tag integration. PCR was performed using flanking genomic and V5-specific primers. The tag-specific PCR fragments (red dashed boxes) were cut out from the gel and sequenced. (**d**,**e**,**i**,**j)** Sequencing of tag-specific PCR fragments.

We used a simplified PCR-based method to produce sgRNA synthesis templates (see Supplementary Protocol for detailed procedures)^7^,^27^,^28^. *In vitro* transcribed sgRNAs were mixed with nCas9n mRNA^10^ and injected into the yolks of zebrafish embryos at the 1-cell stage as described previously^29^, followed by injection of the diluted oligonucleotide. Twenty injected embryos were pooled and DNA was prepared. To test for CRISPR/Cas9 mutagenesis, sgRNA target loci were amplified by PCR, followed by digestion of PCR fragments with BsrGI and BstNI (Fig. 2). We observed nearly complete loss of the BsrGI site at the *tcf21* target site, indicating very efficient mutagenesis (Fig. 2b). Mutagenesis of the *tbx18* target site appeared somewhat less efficient, as indicated by a higher degree of retention of the BstNI restriction enzyme site (Fig. 2g). However, it should be noted that the BstNI restriction enzyme site is offset from the expected CRISPR/Cas9 site by one nucleotide. Only a subset of indels will result in loss of the restriction enzyme site, and thus our RFLP analysis is expected to underestimate the efficiency of the mutagenesis.

To assess for tag integration in injected embryos, we performed PCR reactions using tag-specific/genomic primer pairs. For both loci, we readily obtained PCR bands of the expected sizes (Fig. 2c,h). The PCR fragments were gel-purified and Sanger sequencing was performed directly on the excised PCR fragments to confirm tag integration (Fig. 2d,e,i,j). We observed that the quality of the sequencing significantly drops off near the Cas9 cut site at the 5′ junction between the tag and genomic sequence, but not at the 3′ junction. Since PCR reactions were performed on pools of 20 injected embryos, each PCR fragment is expected to comprise a significant number of independent integration events. This observation therefore suggests a significantly higher rate of indels at the 5′ end of the tag-coding sequence.

### Germline transmission of tag integration events

**Figure 3:**
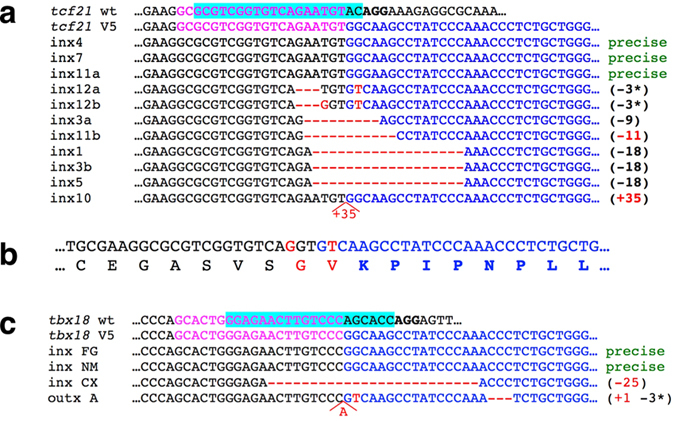
Epitope tag integration events transmitted through the germline. (**a)** An alignment of 5′ junction fragments between tcf21 and V5 tag integration events transmitted through the germline. PAM sequence is in bold, sgRNA target is highlighted in cyan, the homology arm present in the HDR oligo is in magenta, sequence of the V5 tag is in blue, and mismatches are in red. (**b)** DNA and protein sequence of the recovered tcf 21-V5 allele, identical to that shown as inx12b in (**a**). Red indicates nucleotide and/or amino acid changes compared to the expected sequence. (**c)** An alignment of 5′ junction fragments between *tbx18* and V5 tag integration events transmitted through the germline.

After verification of mutagenesis and tag integration, the remaining injected embryos were raised to adulthood, crossed, and tested for germline transmission of tag integration events as well as loss of restriction enzyme sites. Twenty-four fish (twelve incrosses) were tested for germline transmission of mutations and tag integration in *tcf21*. Eight incrosses produced PCR bands indicative of epitope tag integration. The 5′ junctions between *tcf21* and the V5 tag were sequenced (Fig. 3a). At least three F0 fish transmitted perfect integration of the tag (incrosses #4, #7 and #11), and two additional incrosses contained imperfect but likely functional tag integrations: incross #12 (loss of three nucleotides (to be referred to as −3) with or without an additional substitution) and #3 (−9 retaining the core sequence of the V5 tag). Two different PCR products were recovered from incrosses #3, #11 and #12. This could occur if both parents were transmitting tag integration events independently, or if the germline of a single transmitting parent was mosaic for two different integration events.

F1 fish from *tcf21* incrosses #3, #7, #11 and #12 were tail clipped and analyzed for V5 tag integration and/or for indels. None of the twenty-six incross #3 F1 fish screened were positive for the (−9) integration, but six (24%) were positive for an identical (−18) tag integration event, rendering the V5 tag most likely non-recognizable by anti-V5 antibodies. Similarly, none of the fourteen fish from incross #7 were found to have precise tag integration, although two (14%) were positive for an identical (+20) tag integration. F1 fish from incross #11 were not conclusively screened for tag integration. From the incross #12 F1 family, fourteen fish (out of 23 screened) were positive for tag integration by PCR, resulting in germline mosaicism of nearly 61%. Seven were sequenced, and all were found to contain identical near-complete tag integration missing the first codon of the V5 tag and changing one amino acid of the Tcf21 protein (Fig. 3b).

Sixteen F0 fish were screened for germline transmission of tag integration into *tbx18*: six incrosses and four outcrosses. Four of the crosses produced progeny positive for germline transmission of epitope tag integration events, two of them (incross FG and incross NM) with apparent precise integrations (Fig. 3c). Adult F1 fish from the FG family were genotyped for tag integration, and three out of fourteen (21%) contained precise tag integrations.

### Functionality of internally epitope-tagged proteins

Addition of an epitope tag may have a negative impact on translation, folding or stability of a protein, interfere with critical protein-protein interactions, or change intracellular distribution of the protein. Our approach enables parallel recovery of loss of function alleles, and enables testing of the epitope tagged allele by complementation.

**Figure 4:**
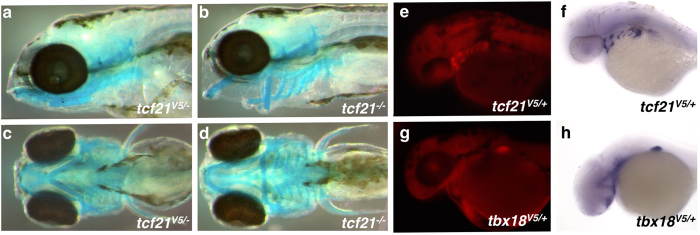
Validation of internally epitope tagged Tcf21 and Tb18. (**a–d**) Internally epitope tagged Tcf21 is fully functional and complements corresponding frameshift mutations. Lateral (**a**,**b**) and ventral (**c**,**d**) views of 5 dpf zebrafish embryos, with cartilage stained with Alcian Blue. Embryos trans-heterozygous for V5-tcf21 and tcf21 frameshift allele (**a**,**c**), and trans-heterozygous for different frameshift alleles (**b**,**d**). (**e–h)** Internally V5 epitope tagged Tcf21 and Tbx18 proteins are detectable by anti-V5 antibodies. Comparison between protein expression as detected by immunohistochemistry using anti-V5 antibodies (**e**,**g**) and mRNA expression detected by whole mount *in situ* hybridization (**f**,**h**).

When screening F1 families for epitope integration and mutations in *tcf21*, we made two observations. First, in many F1 fish both alleles were mutated, consistent with the high degree of mutagenesis observed in injected fish. However, in all cases, one of the alleles was an in-frame deletion or insertion (Supplementary Table 1, Supplementary Fig. 2). Second, at least five of the F1s positive for tag integration harbored frameshift mutations in the second copy of the gene. We proceeded to cross F1s heterozygous for tag integrations to each other as well as other F1 fish, and observed that in each of the crosses where both parents were heterozygous for a frameshift mutation, approximately 25% of the embryos displayed a branchial arch malformation phenotype consistent with the recently published *tcf21* mutant^18^ and morphant^17^ phenotypes (Fig. 4b,d). Together, these observations demonstrate that the Tcf21 protein with an imperfect but near-complete tag integration is functionally wild type.

Similarly, for *tbx18*, we identified two adults trans-heterozygous for a tagged allele and a frameshift allele (Supplementary Table 2). However, incrosses of fish heterozygous for frameshift alleles did not reveal an overt phenotype, and we were able to generate adults homozygous for the (−11) frameshift allele (data not shown and Supplementary Fig. 3). Absence of an overt phenotype is largely consistent with the observation that *tbx18* mutant mice are born alive and relatively normal^30^,^31^, and left us unable to determine if the epitope tagged Tbx18 protein is capable of providing wild type function.

### Internal epitope tags are accessible for anti-V5 antibody binding

We chose to insert the epitope tag into an evolutionarily variable-length segment of the protein based on the assumption that such segments are more likely to be on the surface of the 3-dimensional structure of the protein, and will consequently be accessible for antibody binding even in the native, non-denatured state. We therefore performed whole mount immunohistochemistry using V5 tag-specific antibodies. Internally epitope tagged Tcf21 was readily detectable in an expression pattern consistent with the previously published mRNA expression pattern^15^,^17^,^18^ (Fig. 4e,f). Similarly, internally epitope tagged Tbx18 was readily detectable in developing fin buds, consistent with the mRNA expression pattern (Fig. 4g,h). These results strongly suggest that the internally V5-tagged Tbx18 is able to fold correctly, thus avoiding degradation, and that the V5 epitope is accessible for antibody binding.

## Discussion

Our data demonstrate that a local relative lack of sequence conservation can be used to inform epitope-tagging experiments. The V5 epitope tags, integrated into low-conservation, variable length regions of proteins, are accessible for antibody binding and likely do not significantly interfere with the tertiary structure of the protein. Most significantly, internally V5 epitope tagged Tcf21 is fully capable of providing wild type function. It should also be noted that Tbx18, and especially Tcf21, are much more evolutionarily conserved than the “average” protein. For proteins evolving more rapidly, it may be more appropriate to align orthologs from more closely related taxa. Furthermore, our method does not rely on *a priori* experimental identification of various functional domains in a protein, or on the availability of protein structure data.

The methodology we have developed is very straightforward and does not involve a single cloning step. The amount of time required, from conceiving an experiment to having an established and validated line expressing the protein of interest, is comparable to the best Tol2-based transgenesis methods currently in use^32^,^33^,^34^, and does not suffer from limitations borne by multiple copies of the transgene, position effects and over-, under-, or mis-expression of the protein of interest. Furthermore, parallel recovery of loss-of-function alleles facilitates testing of epitope tagged loci by complementation.

It remains to be seen if our oligonucleotide-based repair strategy can be employed to integrate longer and more repetitive epitope tags such as 3xFLAG, 3xHA or 3xMyc, or the recently described structured tags^35^. However, the sequence conservation-informed principle described here can be applied in conjunction with plasmid-based strategies better suited for integration of larger transgenes, including fluorescent reporters^36^,^37^,^38^.

While we achieved relatively high rates of germline transmission of tag integration and germline mosaicism, there does not appear to be an obvious correlation between the presence of epitope tag integration in the tailclip and in the germline of a given F0 fish (data not shown). Being able to screen F0 fish by tailclip instead of crossing would make our method even more amenable to scaling-up. It may therefore be worthwhile to explore the use of protein-based Cas9^12^, as it may induce editing events earlier in development leading to a higher degree of correspondence between somatic tissues, such as the tail, and the germline.

We also note that the main principle informing tag integration site selection, namely regional lack of protein sequence conservation, can be used to inform epitope integration into other vertebrate genomes, both *in vivo* and *in vitro*^39^.

## Methods

### Approval and Accordance

All described animal experiments were approved by Temple University IACUC committee and carried out in accordance with ULAR guidelines.

### nCas9n mRNA synthesis

pT3TS-nCas9n^10^ was linearized with XbaI and transcribed using the T3 mMessage mMachine *in vitro* transcription kit (ThermoFisher Scientific AM1348). Transcribed mRNA was purified using the Qiagen RNeasy MinElute kit (Qiagen 74204), diluted to 150 ng/μL in RNAse free water (ThermoFisher Scientific AM9937) and 2 μL aliquots were made. Aliquots were stored at −80 °C.

### sgRNA synthesis

We used a cloning-free PCR method similar to ones described previously^7^,^28^ to produce sgRNA synthesis template. We first performed PCR using either Tcf21sg-F1 or Tbx18sg-F1 (Supplementary Table 3) and M13F primers on DR274^40^ template. We then performed agarose gel electrophoresis and purified the bands using GeneJet Gel Extraction kit (ThermoFisher Scientific K0692). Purified DNA was used as the template for the second PCR reaction with sgT7 and sgRNA-R primers. The obtained PCR fragment was purified using GeneJet PCR purification kit (ThermoFisher Scientific K0702) and used as the template for *in vitro* transcription using MEGAshortScript T7 transcription kit (AM1354).

After RNA synthesis, the concentration of sgRNA was assessed by agarose gel electrophoresis. Concentration was estimated by comparing to the RiboRuler RNA ladder (ThermoFisher Scientific SM1833). Unpurified transcription reaction mix was diluted to approximately 60 ng/μL and 8 μL aliquots were made. Aliquots were stored at −80 °C.

### Microinjection

An 8 μL aliquot of sgRNA was thawed and mixed with a 2 μL aliquot of nCas9n mRNA. 3 nL of mix were injected into the yolks of 1-cell zebrafish embryos as described previously^29^.

Appropriate V5 HDR oligonucleotide (Tcf21V5-F1 or Tbx18V5-F1) was diluted to 50 μg/μL in RNAse free water, and 1 nL was injected into the yolks of zebrafish embryos immediately after the RNA injection.

### Testing of CRISPR activity in injected embryos

At 3–5 dpf, 20 embryos were pooled into a standard microcentrifuge tube. DNA was prepared as described previously^41^ and diluted to 100 ng/μL. 1 μL of diluted DNA was used as the template for PCR reactions in 25 μL volume. All PCR reactions were performed using ThermoFisher Scientific Recombinant Taq polymerase (EP0402) and the optional (NH_4_)_2_SO_4_ –containing PCR buffer. Mutations were tested for by determining if there was a loss of the restriction enzyme site: PCR reactions were performed with Tcf21-F1/Tcf21-R1 or Tbx18-F1/Tbx18-R1 primers, and digested as described below. To test for tag integration, PCR was performed with Tcf21-F1/Tbx18-F1 and V5-R1 primers, or with V5-F1 and Tcf21-R1/Tbx18-R1 primers. After agarose gel electrophoresis, the PCR fragments were purified using GeneJet Gel Extraction kit and sequenced using appropriate genomic primers (Tcf21-F1, Tcf21-R1, Tbx18-F1 or Tbx18-F1).

### Loss of Restriction Enzyme Sites

To test for loss of restriction enzyme sites, 10 μL of unpurified PCR reaction was mixed with 15 μL of restriction enzyme mix containing 1.5 μL of appropriate 10X New England BioLabs Buffer (#2+BSA for BsrGI, #3+BSA for BstNI), and 1 μL of restriction enzyme. Restriction enzyme digestion was performed for 4–16 hours.

### Screening for Germline transmission of tag integrations

Embryos from F0 incross or outcross were pooled into batches of 20. Three batches were tested from each pair. DNA was prepared and the first PCR reaction was performed using Tcf21-F1/R1 or Tbx18-F1/R2 primer pairs. The first PCR reaction was diluted 100X in water, and 1 μL was used and the template for the second, nested reaction using Tcf21-F2/Tbx18-F2 with V5-R1 primer, or using Tcf21-R2/Tbx18-R1 with V5-F11. All primers are listed in Supplementary Table 3.

### F1 Genotyping

Adult F1 fish were tail clipped. Genomic DNA was isolated and tested for loss of restriction enzyme site as described above. Testing for tag integration was done by performing PCR using primer pairs Tcf21-F1/V5-R1, Tcf21-R1/V5-F1, Tbx18-F1/V5-R1 or Tbx18-R2/V5-F1. In some instances, V5-F11 was used instead of V5-F1 for F1 genotyping.

### Whole mount immunohistochemistry

Fish heterozygous for V5-Tcf21 or V5-Tbx18 tag integration were outcrossed. 36 hpf embryos were dechorinated then fixed in 4% PFA. All washes were performed in PBT, and embryos were dehydrated then rehydrated through a methanol series as described^42^. Embryos were incubated with primary antibody (ThermoFisher Scientific R960-25), then fluorescent secondary antibody (ThermoFischer Scientific, A-21135), each diluted 1:500 in FBS blocking buffer.

### Whole mount *in situ* hybridization

*In situ* hybridization was performed as previously described^42^. *tcf21* cDNA was amplified using Tcf21-5′UTR-F1 and Tcf21-3′UTR-R2. *tbx18* cDNA was amplified using Tbx18-5′UTR-F1 and Tbx18-R10 (Supplementary Table 3). Each fragment was cloned into pGEM-T vector for transcription.

## Additional Information

**How to cite this article**: Burg, L. *et al*. Internal epitope tagging informed by relative lack of sequence conservation. *Sci. Rep.* **6**, 36986; doi: 10.1038/srep36986 (2016).

**Publisher's note:** Springer Nature remains neutral with regard to jurisdictional claims in published maps and institutional affiliations.

## Supplementary Material

Supplementary Information
