# Supplementary material for: Internal epitope tagging informed by relative lack of sequence conservation
Source: Sci Rep. 2016 Nov 28;6:36986. doi: 10.1038/srep36986 (PMC5125009; doi:10.1038/srep36986)
Supplement: Supplementary Information [file srep36986-s1.pdf]

Supplementary Information for:

Internal epitope tagging informed by relative lack of sequence conservation

Leonard Burg, Karen Zhang, Tristan Bonawitz, Viktorija Grajevskaja, Gianfranco Bellipanni, Richard Waring and Darius Balciunas

Supplementary protocol

Preparation nCas9n mRNA for injection

1. Linearize pT3TS-nCas9n (Jao et al., 2014, PMID:23918387, http://www.addgene.org/46757/) with XbaI
2. *In vitro* transcribe using T3 mMessage mMachine kit (ThermoFisher Scientific #AM1348)
3. Purify the RNA using Qiagen RNeasy MinElute kit (#74204)
4. Dilute 150 ng/uL in RNase- free water (ThermoFisher Scientific, #AM9937)
5. Aliquot to 2uL per eppendorf tube
6. Store aliquots at -80°C.

Target selection

As described the Yeh laboratory (Hwan et al., 2013b, PMID: 23874735) and verified by us, only the NGG PAM site is needed at the target site. The GG dinucleotide required for initiation of transcription by the T7 RNA polymerase can be added to the 5' end of the sgRNA. Furthermore, we find that sgRNAs starting with a single G are sufficiently efficiently transcribed. These simple dinucleotide (NGG) targets are widely abundant in coding sequences. When choosing a target, apply standard principles of primer design, namely 40-50% G/C content, high complexity, and no polypurine/polypyrimidine stretches. If possible select a target where a cut will occur in or near a RFLP-suitable restriction enzyme site. This makes verification of indels straightforward and will make it easy to genotype wild type / heterozygous / homozygous mutant embryos or fish in downstream biological experiments.

Guide RNA synthesis

1. Perform a PCR reaction in 50 μL volume:
   primers: your SG (short guide) forward primer and M13F as the reverse primer
   template: 5 ng of DR274 (Hwan et al., 2013a, PMID:23360964, https://www.addgene.org/42250/)
2. Perform gel extraction (ThermoFisher Scientific, #K0692), elute in 30 μL.

3. Perform a second 50 μL PCR:
   primers: sgT7-F and sgRNA-R
   template: 1 μL of gel-purified PCR 1.

4. Run 5 μL of the PCR on a gel to confirm specific amplification. Perform PCR extraction (ThermoFisher Scientific #K0702) on the remaining 45 μL, elute in 30 μL.

5. Use up to 5 μL as a template for MegaShortscript T7 (ThermoFisher #AM1354) in vitro transcription reaction. The reaction can be performed in 10 μL instead of the 20 μL suggested by the manual. Incubate the reaction at 37°C for 2 hrs.

6. Add 0.5 μL DNAse, incubate for 15 minutes at room temperature. Place the reaction on ice.

Balciunas Lab

7. Perform agarose gel electrophoresis of 1x, 5x and 25x dilutions of the transcription reaction, (equivalent to 1 µL, 0.2 µL and 0.04 µL of the reaction) along with RiboRuler RNA ladder (ThermoFisher Scientific SM1833).

8. Estimate RNA concentration based on the gel. Make several 8 µL aliquots of sgRNA diluted 10X to 100X. Aim for about 60 ng/µl.

Microinjection

1. When you know you have embryos, thaw an 8 µL aliquot of sgRNA and 2 µL aliquot of nCas9n RNA. Add 8 µL of the guide to the 2 µL of nCas9n, mix.

2. Dilute tagging oligonucleotide (20bp homology – TAG - 20bp homology) to 50 ng/µL.

3. Inject 3 nL of mixed RNA into the yolks* of 1-cell embryos.

4. Inject 1 nL of oligonucleptide into the yolks* of embryos.

* These injection volumes and concentrations are optimized for microinjection into the yolk, which we find sufficient for all mutagenesis and transgenesis experiments. They will need to be adjusted if solutions are to be injected into the blastomere.

Post-injection

1. At 1-3 dpf, freeze a batch of 20 embryos for DNA preparation followed by analysis of CRISPR activity by introduced RFLP or Surveyor assay.

2. Raise the remaining embryos to adulthood, screen incross/outcross embryos by nested PCR, 3 batches of 20 from each pair.

Balciunas lab

Supplementary Figure 1. Outline of the epitope tagging and mutagenesis strategy.

Supplementary Figure 2. Assessment of *tcf21* mutant phenotype. a. Incrosses of F1 fish with different *tcf21* mutations produce progeny with jaw malformations. F1 fish from four different families were incrossed. Mutant alleles are designated by the cumulative loss or addition of nucleotides. Two different minus-9 in-frame deletion mutants recovered from two different families are shown as -9a and -9b. * star denotes a stop codon within the insertion. Expected loss-of-function mutants are in shown red. **b.** Alignment of *tcf21* insertion and deletion (indel) mutant sequences. sgRNA target sequence is highlighted in light blue, PAM sequence is in bold. Different mutants are designated by the cumulative change in the length of the nucleotide sequence with expected numbers of nucleotides deleted and added shown in brackets. Missing/additional/mismatched nucleotides are shown in red in the alignment. Microhomologies observed around several of the insertions are underlined. In the +19 mutant, the underlined inserted sequence is reverse complement of the sequence following the Cas9 cut site (5'-CAGGAAAGAGGCGCAAATCAG-3'), part of which is deleted. **c.** Predicted sequences of proteins encoded by *tcf21* indel mutants. Translated nucleotide sequences of wild type and mutant *tcf21*. Nucleotides and amino acids not present in the wild type protein are shown in red.

Supplementary Figure 3. Recovered frameshift mutant of *tbx18*. a. Alignment of wild type and minus-11 mutant *tbx18* DNA sequences. sgRNA target sequence is highlighted in light blue, PAM sequence is in bold. Note that in addition to the 11-nucleotide deletion, the -11 chromosome has an insertion of 3 nucleotides near the end of exon 1. We did not test if this (+3) mutation was present in the founder fish. Exon/intron boundary is shown above. **b.** Predicted sequence of the truncated Tbx18 protein encoded by the -11 mutant. Translated nucleotide sequences of wild type and mutant *tbx18*. Nucleotides and amino acids not present in the wild type protein are shown in red. Boundary between the first and second exon of the predicted cDNA is shown above. Presence of this RNA species was confirmed my sequencing of an RT PCR product (data not shown).

Supplementary Table 1. Genotyping of F1 fish for *tcf21* mutations and V5 tag integration. A summary of the total number of fish that were tail clipped, by family, for analysis of tag integrations/mutations. Some fish were only screened for tag integration or for loss of the restriction enzyme site. The third column lists the number of fish positive for tag integration by PCR / total number screened for tag integration. The fourth column lists the number of fish at least heterozygous for loss of BsrGI restriction enzyme site / total number screened loss of restriction enzyme site. The fifth column lists the number of fish from which both alleles were sequenced and found to harbor CRISPR/Cas9-induced changes. The sixth column lists the genotypes of such fish, with reading frame-preserving alleles in black, frameshift alleles in red, and tag-containing alleles in blue. Asterix (*) indicates a stop codon within the indel. The alleles are further described in **Supplementary Fig. 3**. Note that all six tag PCR-positive fish from incross 3 contained a -18 deletion at the 5' end of the tag, deleting part of the core V5 tag. Both PCR positive fish from incross 7 contained a frame shifting 20 nucleotide insertion at the 3' end

of the tag. Tag integrations in 7 of the 14 PCR-positive F1 fish from the incross 12 family were sequenced, and all were found to a -3 deletion at the 5' end of the tag, removing the first amino acid of the V5 tag but preserving the core. n/d: not conclusively tested for tag integration by PCR.

Supplementary Table 2. Genotyping of F1 fish for *tbx18* mutations and V5 tag integration. A summary of the total number of fish that were tail clipped, by family, for analysis of tag integrations/mutations. All 14 tail clipped F1 fish were analyzed both for tag integration (third column) and for loss of the BstNI restriction enzyme site (fourth column). The fifth column lists the number of fish from which both alleles were sequenced and found to harbor CRISPR/Cas9-induced changes. The sixth column lists the genotypes of such fish, with reading frame-preserving alleles alleles in black, frameshift alleles in red, and tag-containing alleles in blue. All three tag PCR-positive fish had perfect integration of the V5 epitope tag.

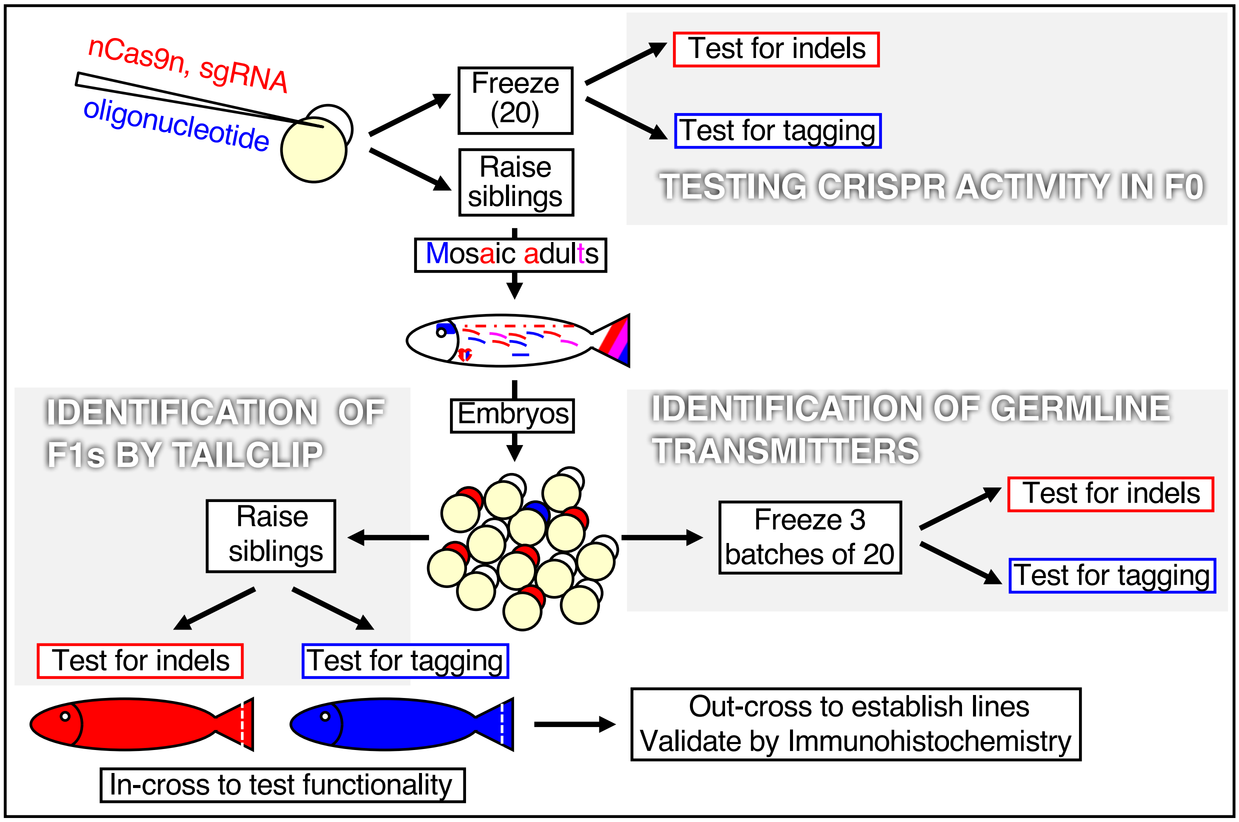

Supplementary Figure 1.

a

Parent 1<br>Genotype (F1 family)	Parent 2<br>Genotype (F1 family)	N<br>Embryos scored	N<br>Embryos with jaw defects	%<br>Embryos with jaw defects
-9a / -10 (inx3)	+15 / +9* (inx3)	92	21	23%
-9b / +16 (inx11)	-9b / +29 (inx11)	60	16	27%
-9b / -55 (inx11)	+3 / +19*(inx7)	132	40	30%
V5 / +5 (inx12)	-9a / -10 (inx3)	86	22	26%
V5 / -11 (inx12)	-9b / +29 (inx11)	114	27	24%
	**Total:**	**484**	**126**	**26%**

b

```
WT tcf21          …AACGAGAGCACGGAGGACAGCTCGAACTGCGAAGGCGCGTCGGTGTCAGAATG          (Cas9 cut site)          TACAGGAAAGAGGCGCAAATCAGCCA…
-9a               …AACGAGAGCACGGAGGACAGCTCGAACTGCGAAGGCGCGTCGGTGTCA-----                                   ----GGAAAGAGGCGCAAATCAGCCA…
-9b               …AACGAGAGCACGGAGGACAGCTCGAACTGCGAAGGCGCGTCGGTGTCAGAA--                                   -------AAGAGGCGCAAATCAGCCA…
-10               …AACGAGAGCACGGAGGACAGCTCGAACTGCGAAGGCGCGTCGGTGTCAGA---                                   -------AAGAGGCGCAAATCAGCCA…
-11 (-12;+1)      …AACGAGAGCACGGAGGACAGCTCGAACTGCGAAGGCGGGTCGGTT--------                                   ---AGGAAAGAGGCGCAAATCAGCCA…
-55               …AACGA------------------------------------------------                                   -------AAGAGGCGCAAATCAGCCA…
+3 (-2;+5)        …AACGAGAGCACGGAGGACAGCTCGAACTGCGAAGGCGCGTCGGTGTCAGAA--                              ACAGGAACAGGAAAGAGGCGCAAATCAGCCA…
+5 (-1;+6)        …AACGAGAGCACGGAGGACAGCTCGAACTGCGAAGGCGCGTCGGTGTCAGAATG                              GACTCCACAGGAAAGAGGCGCAAATCAGCCA…
+9*               …AACGAGAGCACGGAGGACAGCTCGAACTGCGAAGGCGCGTCGGTGTCAGAATG                          AGGCGGAAATACAGGAAAGAGGCGCAAATCAGCCA…
+15 (-1;+16)      …AACGAGAGCACGGAGGACAGCTCGAACTGCGAAGGCGCGTCGGTGTCAGAATG                    GAAAAAGGCGCAAATCACAGGAAAGAGGCGCAAATCAGCCA…
+16 (-1;+17?)     …AACGAGAGCACGGAGGACAGCTCGAACTGCGAAGGCGCGTCGGTGTCAGAATG                   GAAAAGAGGAGGAGGAAAGAGGAGAGAGGCGCAAATCAGCCA…
+19*(-9;+32)      …AACGAGAGCACGGAGGACAGCTCGAACTGCGAAGGCGCGTCGGTGTCAGAATG             CGCCTGATTTGCGCCTCTTTCCTGGGCCAGGTGAGGCGCAAATCAGCCA…
+29 (-1;+30)      …AACGAGAGCACGGAGGACAGCTCGAACTGCGAAGGCGCGTCGGTGTCAGAATGGCACCGGAAAAGAGGAAAGGAAAGAATGGCACAGGAAAGAGGCGCAAATCAGCCA…
```

c

```
WT tcf21          …AACGAGAGCACGGAGGACAGCTCGAACTGCGAAGGCGCGTCGGTGTCAGAATGTACAGGAAAGAGGCGCAAA…
                    N  E  S  T  E  D  S  S  N  C  E  G  A  S  V  S  E  C  T  G  K  R  R  K

-9a               …AACGAGAGCACGGAGGACAGCTCGAACTGCGAAGGCGCGTCGGTGTCA---------GGAAAGAGGCGCAAA…
                    N  E  S  T  E  D  S  S  N  C  E  G  A  S  V  S  -  -  -  G  K  R  R  K

-9b               …AACGAGAGCACGGAGGACAGCTCGAACTGCGAAGGCGCGTCGGTGTCAGAA---------AAGAGGCGCAAA…
                    N  E  S  T  E  D  S  S  N  C  E  G  A  S  V  S  E  -  -  -  K  R  R  K

-10               …AACGAGAGCACGGAGGACAGCTCGAACTGCGAAGGCGCGTCGGTGTCAGAAAGAG…
                    N  E  S  T  E  D  S  S  N  C  E  G  A  S  V  S  E  RGANQPICADQRPMVWPKRASRSRGTLLTRARGRGCACSAKPSPG*

-11 (-12;+1)      …AACGAGAGCACGGAGGACAGCTCGAACTGCGAAGGCGGGTCGGTTAGG…
                    N  E  S  T  E  D  S  S  N  C  E  G  G  S  V  RKEAQISQYAQISAQWCGPRGQAGPEERC*

-55               …AACGAAAGA…
                    N  E  RGANQPICADQRPMVWPKRASRTLLTRARGRGCACSAKPSPG*

+3 (-2;+5)        …AACGAGAGCACGGAGGACAGCTCGAACTGCGAAGGCGCGTCGGTGTCAGAAACAGGAACAGGAAAGAGGCGCAAATCAGCCAATATG
                   N  E  S  T  E  D  S  S  N  C  E  G  A  S  V  S  E  T  G  T  G  K  R  R  K  S  A  N  M

+5 (-1;+6)        …AACGAGAGCACGGAGGACAGCTCGAACTGCGAAGGCGCGTCGGTGTCAGAATGGACTCCACAG…
                   N  E  S  T  E  D  S  S  N  C  E  G  A  S  V  S  E  W  T  P  QERGANQPICADQRPMVWPKRASRSRGTLLTRARGRGCACSAKPSPG*

+9*               …AACGAGAGCACGGAGGACAGCTCGAACTGCGAAGGCGCGTCGGTGTCAGAATGAGGCGGAAATACAG…
                   N  E  S  T  E  D  S  S  N  C  E  G  A  S  V  S  E  *

+15 (-1;+16)      …AACGAGAGCACGGAGGACAGCTCGAACTGCGAAGGCGCGTCGGTGTCAGAATGGAAAAAGGCGCAAATCACAGGAAAGAGGCGCAAATCAGCCAATATG
                   N  E  S  T  E  D  S  S  N  C  E  G  A  S  V  S  E  W  K  K  A  Q  I  T  G  K  R  R  K  S  A  N  M

+16 (-1;+17?)     …AACGAGAGCACGGAGGACAGCTCGAACTGCGAAGGCGCGTCGGTGTCAGAATGGAAAAGAGGAGGAGGAAAGAGGAG…
                   N  E  S  T  E  D  S  S  N  C  E  G  A  S  V  S  E  W  K  R  G  G  G  K  R  REAQISQYAQISAQWCGPRGQAGPEERC*

+19*(-9;+32)      …AACGAGAGCACGGAGGACAGCTCGAACTGCGAAGGCGCGTCGGTGTCAGAATGCGCCTGATTTGCGCCTCTTTCCTGGGCCAGGTGAGGCGCAAATCAGCCAATATGC
                   N  E  S  T  E  D  S  S  N  C  E  G  A  S  V  S  E  C  A  *

+29 (-1;+30)      …AACGAGAGCACGGAGGACAGCTCGAACTGCGAAGGCGCGTCGGTGTCAGAATGGCACCGGAAAAGAGGAAAGGAAAGAATGGCACAG…
                   N  E  S  T  E  D  S  S  N  C  E  G  A  S  V  S  E  W  H  R  K  R  G  K  E  R  M  A QERGANQPICADQRPMVWPKRASRSRGTLLTRARGRGCACSAKPSPG*
```

Supplementary Figure 2

a

```
                                                                            exon 1-><-intron 1
WT tbx18     AGTCCGCAACCCAGCACTGGGAGAACTTGTCCCAGCACCAGGAGTTGCGAGATAGATTGC---ACCAGCGACGAATCCCGTAAGT
-11          AGTCCGCAACCCAGCACTGGGAGAACTTGTC-----------AGTTGCGAGATAGATTGCAGAACCAGCGACGAATCCCGTAAGT
             *******************************           ******************   **********************
```

b

```
                                                                          exon 1-><-exon 2
WT tbx18     AGTCCGCAACCCAGCACTGGGAGAACTTGTCCCAGCACCAGGAGTTGCGAGATAGATTGCACCAGCGACGAATCCCCGGAGCCTGA
              S  P  Q  P  S  T  G  R  T  C  P  S  T  R  S  C  E  I  D  C  T  S  D  E  S  P  E  P

                                                                   exon 1-><-exon 2
-11          AGTCCGCAACCCAGCACTGGGAGAACTTGTCAGTTGCGAGATAGATTGCAGAACCAGCGACGAATCCCCGGAGCCTGA
              S  P  Q  P  S  T  G  R  T  C  Q  L  R  D  R  L  Q  N  Q  R  R  I  P  G  A  *
```

Supplementary Figure 3

F1 family	F1 tailclipped	tag PCR-positive	RFLP-positive	confirmed transheterozygtes	confirmed genotypes
Incross 3	31	6/26 (all -18, incomplete)	25/25	2	(-9/-10), (+15/+9-->stop)
Incross 7	14	2/14 (both +20)	13/14	1	(+3/+19), 2x (wt/tag+20)
Incross 11	11	n/d	11/11	2	(wt/-56), (-9/+29), (-9/+16)
Incross 12	23	14	14/14	7	(tag-3/+19), (tag-3/-11), 3x(tag-3/+5), 2x(tag-3/nd)

Supplementary Table 1. Genotyping of F1 fish for tcf21 mutations and V5 tag integration.

F1 family	F1s tailclipped	tag PCR-positive	RFLP-positive	sequenced transheterozygtes	transheterozygous genotypes
FG	14	3	7/14	4	(tag/-18), (tag/-8), (tag/-22), (-18/-11)

Supplementary Table 2. Genotyping of F1 fish for tbx18 mutations and V5 tag integration.

Supplementary Table 3. Sequences of oligonucleotides used in this study.

Oligo-nucleotide	Sequence 5’ to 3’
Tcf21sg-F1	CGCTAGCTAATACGACTCACTATA**GCGTCGGTGTCAGAATGTAC**GTTTTAGAGCTAGAAATAG
Tbx18sg-F1	CGCTAGCTAATACGACTCACTATA**GGAGAACTTGTCCCAGCACC**GTTTTAGAGCTAGAAATAG
Tcf21V5-F1	GCGCGTCGGTGTCAGAATGT**GGCAAGCCTATCCCAAACCCTCTGCTGGGCCTGGACTCCACA**GGAAAGAGGCGCAAATCAGC
Tbx18V5-F1	GCACTGGGAGAACTTGTCCC**GGCAAGCCTATCCCAAACCCTCTGCTGGGCCTGGACTCC**ACCAGGAGTTGCGAGATAGA
sgT7	GCTAGCTAATACGACTCACT
sgRNA-R	AAAAGCACCGACTCGGTG
M13F	GTAAAACGACGGCCAGT
Tcf21-F1	ACAAGTTATGCTCATCCTCAGCTCG
Tcf21-F2	TTCCACATAGCCAGTTGCTTCATCT
Tcf21-R1	CATATTTGTCATTGGCGAGTATCTG
Tcf21-R2	CGCAGAGTGTCCAGCTTGGAGAGTT
Tcf21-5’UTR-F1	CTCCACGTCCAGTCAGAGAACCTC
Tcf21-3’UTR-R2	AACAAAGGCGCGTGACATGA
Tbx18-F1	CGTTGACAGACCGATGAGACTGTT
Tbx18-F2	TCTCGGTCGGTAACCATGGCTGAA
Tbx18-R1	CGTTATGCGTAACTTATTGTCGACA
Tbx18-R2	CTCTGTTACCCTGACTTTACCTAAG
Tbx18-5’UTR-F1	GTTGACAGACCGATGAGACT
Tbx18-R10	TGGAGTGCAGAATGATATGGCC
V5-F1	ATGTGTGGCAAGCCTATCCCAAAC
V5-F11	GGCAAGCCTATCCCAAACCCTCTG
V5-R1	TGTGGAGTCCAGGCCCAGCAGA
